# Identifying musculoskeletal conditions in electronic medical records: a prevalence and validation study using the Deliver Primary Healthcare Information (DELPHI) database

**DOI:** 10.1186/s12891-019-2568-2

**Published:** 2019-05-03

**Authors:** Bridget L. Ryan, Heather L. Maddocks, Scott McKay, Robert Petrella, Amanda L. Terry, Moira Stewart

**Affiliations:** 1 Centre for Studies in Family Medicine, Department of Family Medicine, Department of Epidemiology & Biostatistics, Schulich School of Medicine & Dentistry, The University of Western Ontario, 1151 Richmond Street, London, Ontario N6A 3K7 Canada; 2 Centre for Studies in Family Medicine, Department of Family Medicine, Schulich School of Medicine & Dentistry, The University of Western Ontario, 1151 Richmond Street, London, Ontario N6A 3K7 Canada; 3 School of Kinesiology, Department of Family Medicine, Schulich School of Medicine & Dentistry, The University of Western Ontario, 1151 Richmond Street, London, Ontario N6A 3K7 Canada; 4 Centre for Studies in Family Medicine, Department of Family Medicine, Department of Epidemiology & Biostatistics, Schulich Interfaculty Program in Public Health, Schulich School of Medicine & Dentistry, The University of Western Ontario, 1151 Richmond Street, London, Ontario N6A 3K7 Canada; 5 Centre for Studies in Family Medicine, Schulich School of Medicine & Dentistry, 1151 Richmond Street, London, Ontario N6A 3K7 Canada

**Keywords:** Musculoskeletal conditions, Electronic medical records, Validation, Health administrative data, International classification of primary care, International classification of diseases, DELPHI database

## Abstract

**Background:**

Musculoskeletal (MSK) conditions are a common presentation in primary care. This study sought to determine the prevalence of MSK conditions in primary care in Ontario and to validate the extent to which health administrative date billing codes accurately represent MSK diagnoses.

**Methods:**

De-identified electronic medical records (EMR) from the DELPHI database in southwestern Ontario, which contains 2493 patients (55.6% female, mean age 50.3 years (SD = 22.2)) and 21,964 encounters (July 1, 2006-June 30, 2010) were used for the analyses. Outcomes included: validation measures of agreement between International Classification of Diseases (ICD-9) diagnostic codes (health administrative data) and International Classification of Primary Care (ICPC) diagnoses defined as the reference standard, time to first ICD-9 code, prevalence, and healthcare utilization.

**Results:**

There were 2940 true positive MSK encounters with primary care practitioners for 998 patients. Performance of the ICD-9 diagnostic codes included sensitivity = 76.5%, specificity = 95.2%, PPV = 94.6%, and NPV = 78.7%, compared to the ICPC reference standard. The majority of 998 patients were coded with both an ICPC and ICD-9 MSK code at their first or second encounter (67.4%). However, 23.5% of patients with the ICPC reference standard MSK were never coded with ICD-9. Four-year prevalence of MSK was 52.3% and varied by age (4.5% 0-17 years, 20.1% 18–44, 42.7% 45–64, and 32.7% 65+). Patients at MSK encounters had a higher number of: investigations (17.9% compared to 9.1%, *p* < .0001); referrals (17.6% compared to 14.3%, *p* < .0001); and prescriptions for opioids (17.2% compared to 5.3%, *p* < .0001).

**Conclusions:**

This study determined the prevalence of musculoskeletal conditions in primary care in Ontario using a reference standard definition. The study highlighted the value of using primary care ICPC codes to validate a definition for musculoskeletal conditions. Health administrative data can be used to ascertain the presence of musculoskeletal conditions; however, ICD-9 codes may underrepresent the prevalence of MSK conditions.

**Electronic supplementary material:**

The online version of this article (10.1186/s12891-019-2568-2) contains supplementary material, which is available to authorized users.

## Background

Musculoskeletal conditions are a common presentation among patients in primary care [1], yet their etiology, prevention and treatment remain poorly understood [2]. The prevalence of musculoskeletal conditions starts early in life and increases with age [3]. Musculoskeletal conditions are a major driver of health care utilization and health care costs [1, 4]. This intensive use of resources does not necessarily translate to improved patient outcomes; for example, recurrence rates of low back pain range from 24 to 80% [5, 6].

Canada’s health care system provides universal, single-payer, government-funded medical care including primary care. In Ontario, health administrative data offer rich and plentiful information about patients’ health problems and their health care utilization; many conditions have been studied using health administrative data [7]. Health administrative data presents an opportunity to conduct surveillance and research on musculoskeletal conditions, allowing us to identify and characterize those with musculoskeletal conditions, to measure their health care utilization and associated costs, all without the necessity of primary data collection. However, currently we do not have standardized definitions or algorithms to identify musculoskeletal conditions in health administrative data. Definitions and algorithms need to be developed and validated to make these administrative data useful for research purposes. Much of the care for musculoskeletal conditions occurs in primary care (as compared to hospital care, for example) and so it is particularly important that definitions and algorithms are developed which use primary care data. For example, in Ontario where this study took place, a patient may present and discuss multiple conditions with their family physician, but only one diagnostic code will be recorded for billing purposes to the Ontario Health Insurance Program (OHIP). It is only this code that becomes a component of health administrative data. Validation work is therefore necessary to determine to what extent these billing codes accurately represent musculoskeletal diagnoses.

The first objective of this study was to determine the prevalence of musculoskeletal conditions in primary care patients, who were characterized according to their demographic and clinical characteristics, including healthcare utilization. The second study objective was to measure the extent to which OHIP diagnostic codes for musculoskeletal conditions (the health administrative data used for billing a primary care in-office encounter) agreed with the separate set of codes defined as the reference standard. We hypothesized that, compared to the reference standard, OHIP diagnostic codes would demonstrate moderate sensitivity and high specificity for musculoskeletal conditions.

## Methods

### Setting

This retrospective cohort study used de-identified data from the DELPHI (Deliver Primary Healthcare Information) Electronic Medical Record (EMR) database. This database is located at the Centre for Studies in Family Medicine at Western University, London, Ontario, Canada and contains 29,303 patient records from 23 participating family physicians at 10 practice sites across the southwestern region of the province of Ontario [8]. The age and sex of patients in the DELPHI database have been compared to the Canadian census, and are approximately representative of the population of Ontario [9]. Ethics approval for the DELPHI project was received from Western University’s Ethics Review Board (reference number 11151E).

### Participants

In October 2005, physicians participating in the DELPHI project were asked to use the International Classification of Primary Care (ICPC) to code encounter diagnoses for a random selection of one patient per day until a maximum of 10% of their patients were prospectively coded as part of the research study [10]. The physicians who agreed to code a subset of their patients for research purposes were trained in the use of ICPC and provided with ongoing support. ICPC is an internationally accepted classification system specifically suited to the practice environment of primary healthcare [11]. A total of 3168 patients were coded with ICPC, contributing to an ICPC coded cohort of primary care patients that is not found elsewhere in Canada.

For this study, 4 years of data were extracted between July 1st, 2006 and June 30th, 2010. Of the ICPC cohort, 2493 patients were eligible for inclusion in our study by having at least one encounter with both ICPC coding and an International Classification of Diseases version 9 (ICD-9) diagnostic code recorded for the Ontario Health Insurance Program billing. These patients had at total of *n* = 21,964 encounters coded by 17 physicians at seven practice sites.

### Selection of ICPC codes as the reference standard for diagnosis of musculoskeletal conditions

The presence of a musculoskeletal ICPC symptom or diagnosis code recorded by the physician in the EMR at the end of a patient encounter was considered the reference standard for this study (hereafter referred to as the ‘ICPC reference standard’). A list of ICPC codes for musculoskeletal conditions were selected and approved by two clinician researchers (SM, RP) through consensus. All of the symptoms and diagnoses in the Musculoskeletal Chapter were selected as the ICPC reference standard to identify patients with musculoskeletal conditions [11]. Four additional codes from other chapters were added because of their inclusion in the selected ICD-9 list: N93 Carpal tunnel syndrome (Neurological Chapter), S20 Corn/callosity, S94 Ingrown nail (Skin Chapter), and T92 Gout (Metabolic/Endocrine Disorders Chapter). A total of 62 ICPC codes were selected as the reference standard, and only one code was necessary to identify patients as having a musculoskeletal condition.

### Selection of ICD9 codes

Congruent with the selection of the ICPC list of musculoskeletal conditions, the list of OHIP ICD-9 diagnostic billing codes (hereafter referred to as the ‘ICD-9 billing codes’) was also developed through a consensus process. The Ontario Ministry of Health and Long Term Care’s (2016) Resource Manual for Physicians contains an alphabetical list of diagnoses with three digit ICD-9 codes that was used to identify musculoskeletal conditions [12]. All codes from the ‘Diseases of the Musculoskeletal System and Connective Tissue’ section were selected. In addition, the following codes were selected if they would be resolved by treatment as an musculoskeletal condition: 015 (Tuberculosis of the joint), 099 (Duschenne’s Disease), 274 (Gout), 359 (Muscular Dystrophy), 370 (Keratitis of Joint), 376 (Keratoconus of Joint), 524 (Meporomandibular Joint Disorders), 700 (Calluses), 701 (Keloid of Joint), 703 (Ingrown Nail), Fractures (802, 803, 805–808, 810, 812–816, 821, 823, 824, 829 and 832), Dislocations (831, 834, and 839) and Sprains (840, 842, 844, 845, 847, 848). A total of 62 ICD-9 billing codes were selected.

### Patient characteristics

The following patient characteristics were extracted: sex (male/female), age in years, age groups (18–44, 45–64, 65+ years), patient’s rural/urban residence using the first three digits of their postal code (where if the second character was 0 they were classified as rural, and if the second character ranged from 1 to 9 they were urban [13].

### Healthcare utilization measures

For each patient, visits to the family physician were captured. For each encounter, the investigations ordered, referrals to specialists and prescriptions for opioids were captured. Opioid name brand and generic names were identified using the Canadian Compendium of Pharmaceuticals and Specialties [14].

### Analyses

A descriptive analysis of the prevalence of musculoskeletal conditions (using the ICPC reference standard) and a comparison of the characteristics of patients with and without musculoskeletal conditions was done using Pearson Chi-Square statistics for categorical factors and using ANOVA for the continuous factor of age in years using a significance level of *p* < .05. The prevalence of the top twenty musculoskeletal conditions recorded by ICPC and ICD-9 was calculated.

For the validation, the ICPC reference standard was compared to the ICD-9 billing codes to validate the extent to which the ICD-9 billing code represented the prevalence of musculoskeletal conditions. This validation analysis was conducted using two approaches. First, the *patient* was the unit of analysis where the first occurrence of an ICPC reference standard code was captured for each patient. Then, the presence of an ICD-9 billing code was identified at either the same encounter or at an encounter subsequent within the 4 years of data. The second approach used the *encounter* as the unit of analysis where the occurrence of an ICPC reference standard and ICD-9 billing code were captured for each individual encounter. For each of these two approaches, sensitivity, specificity, positive predictive value and negative predictive value were calculated as performance measures.

The total number of visits and mean number of months it took for a patient to receive their first ICD-9 billing code after an ICPC reference standard code were calculated. The remainder of patients (without an ICD-9 billing code) were assessed for their distribution across practices and physicians in the database to assess data quality and observing whether there was only one physician or practice who did not use any ICD-9 billing codes for musculoskeletal conditions. In addition, the amount of time subsequent to the last ICPC reference standard visit was measured to determine if patients had additional visits in the database where they would have an opportunity to receive an ICD-9 billing code.

The mean number of visits to the family physician was calculated for patients with and without musculoskeletal conditions. Healthcare utilization was compared across encounters using investigations ordered, referrals made and opioid prescriptions. For investigations and referrals, overall utilization was measured as well as the proportion of investigations and referrals related to musculoskeletal conditions. Overall opioid prescriptions were measured. For all three utilization measures, encounters for patients with and without musculoskeletal conditions (using the ICPC reference standard) were compared as a whole and by age group using Pearson Chi-square testing. The proportion of encounters with a referral to each type of medical specialty was calculated.

All analyses were performed in SPSS Version 24 [15].

## Results

Of the 2493 patients in the selected cohort, 1305 were identified as having musculoskeletal conditions (defined with the ICPC reference standard). Table 1 describes the sample, comparing characteristics of patients with and without musculoskeletal conditions. There was no significant difference by sex, with the overall sample being 55.6% female. Patients with musculoskeletal conditions were significantly older, at 55.5 years compared to patients without musculoskeletal conditions at 44.6 years. The study cohort was predominantly urban and there was no significant difference between patients with and without musculoskeletal conditions by location.

**Table 1:** Characteristics of patients (*n* = 2493)

		Patients with Musculoskeletal Conditions^a^ (*n* = 1305)		Patients without Musculoskeletal Conditions (*n* = 1188)		
		# Patients	% Patients	# Patients	% Patients	*P* value
Sex						
	Males	583	44.7	523	44.0	.775
	Females	722	55.3	665	56.0	
Age Group						
	0–17	59	4.5	194	16.3	<.001
	18–44	262	20.1	337	28.4	
	45–64	557	42.7	394	33.2	
	65+	427	32.7	263	22.1	
Age in years, mean and Standard Deviation		55.5	19.2	44.6	23.9	<.001
Location^b^						
	Urban	1012	78.9	891	76.9	.254
	Rural	270	21.1	267	23.1	
	Missing	23		30		

^a^Reference Standard for identifying patients with musculoskeletal conditions is if they had 1+ International Classification of Primary Care coded encounters with a diagnosis code for a musculoskeletal condition at any time in the database

^b^Patient location measured using first three digits of postal code. Numeral of 0 = rural, and 1–9 = urban

The prevalence of musculoskeletal conditions was 52.3% across the 4 years of the cohort. In any given year, the prevalence ranged from 36.5 to 43.0% of visiting patients having at least one encounter for musculoskeletal conditions (results not shown). Additional file 1: Tables S1 and S2 shows the top twenty musculoskeletal conditions coded in ICPC and ICD-9. In Table 2, Section A, the performance characteristics of the validation are reported at the patient level. The sensitivity was moderate, at 76.5% overall, and varied by age (ranging from 71.7–79.4%). Specificity was high overall at 95.2%, and varied by age (ranging from 93.9–100.0%). The positive predictive value was high at 94.6% overall, and varied by age (ranging from 91.2–100.0%). However, the negative predictive value was lower at 78.7% (ranging from 67.4–92.4 by age). For the encounter level (see Table 2, Section B), sensitivity was lower than the patient level at 57.0 and also varied by age, with the sensitivity being the highest in the youngest age group (69.6) and decreased with age to a low of 51.0 for those 65+ years. The specificity was also high at 97.5 and varied little by age (97.5–100.0). The positive predictive value was lower than the patient level at 87.3 and the negative predictive value was higher at 88.1.

**Table 2:** Performance characteristics of ICD-9 based validation for musculoskeletal conditions against the ICPC Reference Standard

	Total	TP	TN	FN	FP	Sensitivity [95 CI%]	Specificity [95 CI%]	PPV [95 CI%]	NPV [95 CI%]
A. Analysis at Level of Patient									
All Ages	2493	998	1131	307	57	76.5 (74.1–78.8)	95.2 (93.8–96.4)	94.6 (93.1–95.8)	78.7 (77.0–80.3)
0–17	253	43	194	16	0	72.9 (59.7–83.6)	100.0 (98.1–100.0)	100.0 (100.0–100.0)	92.4 (88.9–94.9)
18–44	599	207	317	55	20	79.0 (73.6–83.8)	94.1 (91.0–96.3)	91.2 (87.1–94.1)	85.2 (82.0–88.0)
45–64	951	442	370	115	24	79.4 (75.8–82.6)	93.9 (91.1–96.1)	94.9 (92.6–96.5)	76.3 (73.2–79.1)
65+	690	306	250	121	13	71.7 (67.1–75.9)	95.1 (91.7–97.3)	95.9 (93.3–97.6)	67.4 (63.9–70.7)
B. Analysis at Level of Encounter									
All Ages	21,964	2940	16,378	2220	426	57.0 (55.6–58.3)	97.5 (92.2–97.7)	87.3 (86.2–88.4)	88.1 (87.7–88.4)
0–17	1133	55	1054	24	0	69.6 (58.3–79.5)	100.0 (99.7–100.0)	100.0 (100.0–100.0)	97.8 (96.9–98.4)
18–44	4322	600	3316	314	92	65.6 (62.5–68.7)	97.3 (96.7–97.8)	86.7 (84.1–88.9)	91.3 (90.6–92.3)
45–64	8688	1326	6214	961	187	58.0 (55.9–60.0)	97.1 (96.6–97.5)	87.6 (86.0–89.1)	86.6 (86.0–87.2)
65+	7821	959	5794	921	147	51.0 (48.7–53.3)	97.5 (97.1–97.9)	86.7 (85.7–88.5)	86.3 (85.7–86.8)

*TP* True Positive, *TN* True Negative, *FN* False Negative, *FP* False Positive, *CI* Confidence Interval, *PPV* Positive Predictive Value, *NPV* Negative Predictive Value

Table 3 reports the time to diagnosis for patients. Of the 1305 patients with musculoskeletal conditions (using the ICPC reference standard), 998 were true positives (i.e., with an ICD-9 billing code). Of these, over half (55.2%) were coded with an ICD-9 billing code at their first visit, 12.2% at their second visit (mean 8.3 months), and approximately 9.5% at their 3+ visit (average ranging from 13 to 19.5 months). The remaining 307 patients (23.5%) were false negatives; i.e., they never received an ICD-9 billing code at any time in our 4 year database.

**Table 3:** Time to first ICD-9 Musculoskeletal Code at Encounter with ICPC Reference Standard Code (*n* = 1305 patients)

		# Patients	% Patients	Cumulative %	Mean Months^a^	Standard Deviation
Total number of patients with an ICPC Reference Standard Coded Encounter		1305	100.0			
Number of Visits to ICD-9 Code	1st Visit	720	55.2	55.2	n/a	
	2nd	159	12.2	67.4	8.3	8.9
	3rd	70	5.4	72.7	13.0	10.8
	4th	24	1.8	74.6	12.5	9.1
	5th+	25	1.9	76.5	19.5	8.8
Remainder of patients without an ICD-9 Musculoskeletal Code		307	23.5		n/a	

^a^Mean number of months between first ICPC Musculoskeletal coded encounter and first ICD-9 Musculoskeletal coded encounter

A further examination of the 307 false negative patients found that they were distributed across practices and physicians; i.e., there was no one practice or physician responsible for the majority of false negatives. In addition, we examined the number of visits these 307 patients had following their first ICPC reference standard encounter to ensure there was sufficient opportunity for the recording of ICD-9 billing codes. The vast majority (66.4%) had five or more subsequent visits where they could be coded with an ICD-9 billing code. A minority (6.2%) had no visits after their first ICPC reference standard coded visit, which left them only the one visit in which to receive an ICD-9 billing code.

**Fig. 1:**
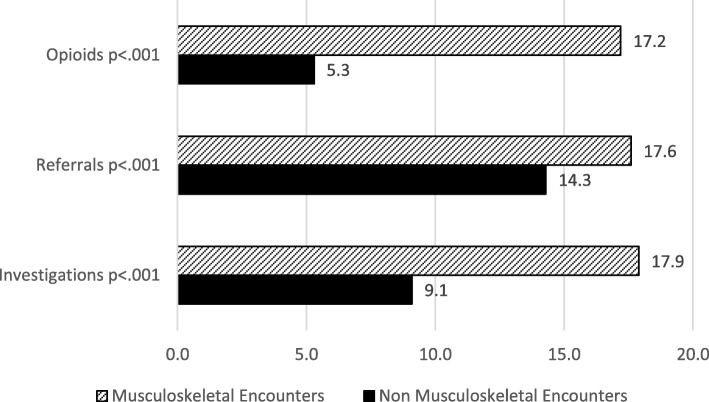
Healthcare utilization at ICPC Reference Standard Musculoskeletal Coded Encounters Compared to Non Musculoskeletal Coded Encounters (*n* = 21,964): percentage of visits with 1+ opioids prescribed, referrals or investigations

In terms of healthcare utilization, patients with musculoskeletal conditions had a significantly higher mean of 3.1 family physician visits per year (standard deviation 3.1) compared to patients without musculoskeletal conditions (mean 1.2, standard deviation 1.6) (results not shown in a table). Fig. 1 shows the proportion of encounters for musculoskeletal conditions (defined using the ICPC reference standard) with investigations ordered, referrals made and opioids prescribed compared to the healthcare utilization for the remainder of encounters. For all three measures, healthcare utilization was significantly higher at encounters for musculoskeletal conditions. Investigations were almost 9 % higher, referrals were 3.3% higher and opioids were 12% higher at encounters for musculoskeletal conditions. Patterns were similar by age group (results not shown) for musculoskeletal related investigations and referrals, however, the pattern for opioid use varied by age. No patients 0–17 years were prescribed opioids and of those over 18 years, the proportion varied significantly by age group with 26.1% of those 18–44 years, 18.4% in 45–64 years, and 12.1% for patients 65+ years (*p* < .0005). The most frequent referrals for musculoskeletal and non-musculoskeletal encounters are shown in Additional file 2: Table S3.

## Discussion

This study set out to determine the prevalence of musculoskeletal conditions in primary care patients, and health care utilization for those with musculoskeletal conditions compared to those without. Using a reference standard definition, this study found a high overall prevalence of musculoskeletal conditions (52%). Prevalence increased in each subsequent age group until 64 years where it dropped for those 65 plus years. Prevalence was calculated over a four-year period and would be expected to be higher than annual prevalence rates reported in the literature. Annual rates were also calculated, and over 4 years the lowest rate was 36.5%.

For the purposes of comparison, few studies were identified that provided prevalence estimates for musculoskeletal conditions overall. Studies varied with respect to the study population and with specific kinds or subsets of musculoskeletal conditions. For example, an Ontario study that examined musculoskeletal conditions overall in the general population using health administrative data found a prevalence of 22.3% per year [1]. A study of primary care patients in Germany found a rate of low back pain of 49.5%, however this was in a population with multimorbidity [16]. A Statistics Canada publication reported a prevalence of chronic pain of 22% but the study did not specify if the pain was from musculoskeletal conditions [17]. Some studies examined only arthritic musculoskeletal conditions and so naturally found much lower prevalence [18].

This current study also determined that patients with musculoskeletal conditions had more visits to the family physician and at those visits they received more investigations, more referrals, and more prescriptions for opioids than those without musculoskeletal conditions. The one other Ontario study regarding health care utilization [1] also found a high economic burden of musculoskeletal conditions as the majority of care for persons with musculoskeletal conditions was delivered in primary care rather than hospitals. Specifically, MacKay et al. (2010) found a higher rate of referrals for patients with musculoskeletal conditions at 33% [1]. This is higher than our rate of 17.9%. However, this is because our rate is calculated as the proportion of encounters for patients with musculoskeletal conditions with a referral, whereas MacKay et al. reported the proportion of patients with musculoskeletal conditions who saw a specialist at least once in a 1 year period, allowing patients to have a whole year to be counted as having contact with a specialist. Our study is measuring individual encounters with a referral. Both rates however demonstrate the burden of musculoskeletal conditions on both primary care and the health system as a whole.

This study validated one definition for musculoskeletal conditions in primary care, both acute and chronic; by comparing the reference standard to the diagnostic billing codes using ICD-9. In Canada, these ICD-9 codes are often the only diagnostic billing codes available in EMR data and so are the only method for identifying patients with musculoskeletal conditions in primary care EMR data and in health administrative data. The definition for musculoskeletal conditions using ICD-9 codes had good sensitivity overall (76.5) and across age groups. Sensitivity was higher for those between the ages of 18 and 64, compared to the young and the old age groups. The proportion of patients who were false negatives increased in each age group from 6.3% of 0–17 year olds to 17.5% of those 65+ years. This variation by age may be due to the fact that older patients may be presenting for other conditions at their visits and these may be the conditions the physician chooses for the diagnostic billing code. The study found half of the patients with musculoskeletal conditions received a musculoskeletal diagnosis at the first visit, and approximately another quarter receiving a diagnosis in two or more visits. The remaining 25% never received a diagnosis for musculoskeletal conditions using ICD-9 codes.

Future research will validate definitions for acute and chronic musculoskeletal conditions separately. The ICD-9 codes for musculoskeletal conditions do not always make distinctions between what are acute musculoskeletal and what are chronic musculoskeletal conditions. As an example, there is one code for ‘knee sprain/pain’. This code could be used for an acute sprain or for on-going knee pain. Additionally, acute conditions can continue over time and move from being acute to either resolving or to continuing on to a chronic condition. Therefore, future research will use an episode of care approach that considers the length of time for particular musculoskeletal conditions to move from acute to chronic.

The data for this study are from 2006 to 2010; this suggests potential limitations. First, it is possible that there has been a change in coding behavior since that time. Within our own DELPHI database, we did find an initial improvement in coding behavior from its inception in October 2005 to March 2006 as physicians were trained in coding [10]. For this reason, we only used data beginning in March 2006. We found no evidence that speaks directly to changes in coding behavior over time; however, we did identify studies that spoke of the need to intervene in order to improve coding [19, 20]. This may suggest that, in the absence of interventions, it is unlikely coding behavior would have changed substantially over time. There will be, of course, variation among providers in how they choose to code certain encounters and diagnoses; we would not expect perfect agreement. However, it is reasonable to think that, for the ICPC codes, if a patient was seen for *any* musculoskeletal condition, the physician would have coded some variation on a musculoskeletal condition. We had two physicians confirm the list of *all* acceptable musculoskeletal conditions to ensure all musculoskeletal conditions coded by ICPC would be captured. The motivation for the study was to determine the extent to which physicians did capture musculoskeletal conditions in health administrative data. A second important consideration is that the use of ICD-9 codes is decreasing. In the United States, for example, the use of ICD-10 has been mandated since 2015 [21]. While hospitals have moved to ICD-10 codes in Canada, ICD-9 is still being used in many Canadian jurisdictions for primary care where much of the care for musculoskeletal conditions occurs [22, 23]. This means that, for some time to come, retrospective studies using health administrative data will need validated definitions of conditions with ICD-9. As well, this study suggests methods that can be used in the future to validate ICD-10 codes using the robust primary care ICPC codes as a reference standard.

In Ontario, physicians send only one ICD-9 billing code to the provincial health insurance system. This one code becomes part of the health administrative data for the province. Because primary care visits often include the discussion of multiple issues, it is expected that musculoskeletal conditions would not get coded for all visits where they are discussed. Other billing codes may be chosen by the physician for various reasons such as the relative importance of the condition to the patient’s health or the reimbursement schedule for particular conditions. This is why we examined multiple visits to determine at what point musculoskeletal conditions were coded. Other health systems may have the ability to code multiple billing codes and, for those contexts, it might be expected that the sensitivity of one billing code may be higher.

An additional limitation of the cohort used in this study is the small sample size relative to the Ontario health system. The results may not be generalizable to the entire province; however, the primary care practices in the DELPHI database contain urban and rural practices, and the patient characteristics are similar to that of the Ontario population [24]. As well, a minority (6.2%) of patients with ICPC reference standard code but without an ICD-9 billing code (false negatives) had only one subsequent visit within the study time period in which to receive an ICD-9 code. It is possible, therefore, that the musculoskeletal condition may have been coded after the study end date.

## Conclusions

This study determined the prevalence of musculoskeletal conditions in primary care in Ontario using a reference standard definition, and described the health care utilization for these conditions. The study highlighted the value of using primary care ICPC codes to validate a definition for musculoskeletal conditions. Future studies could use this method to validate musculoskeletal conditions coded with ICD-10. Retrospective studies, where ICD-9 codes are still prevalent, can use this validated definition for research concerning musculoskeletal conditions in both primary care EMR data and population-based health administrative data. It is important to note that the prevalence of musculoskeletal conditions using only ICD-9 codes was under-estimated.

## Additional files

Additional file 1: **Table S1.** Prevalence of the Top 20 ICPC Coded Musculoskeletal Conditions (*n* = 2493 patients). **Table S2.** Prevalence of the Top 20 ICD9 Coded Musculoskeletal Conditions (*n* = 2493 patients) (DOCX 14 kb)

Additional file 2: **Table S3.** Most Frequent Referrals at ICPC Reference Standard Musculoskeletal Coded Encounters Compared to Non Musculoskeletal Encounters (DOCX 13 kb)

## Abbreviations

CI: Confidence Interval
DELPHI: Deliver Primary Healthcare Information
EMR: Electronic Medical Record
FN: False Negative
FP: False Positive
NPV: Negative Predictive Value
PPV: Positive Predictive Value
TN: True Negative
TP: True Positive
