# Supplementary material for: Identifying musculoskeletal conditions in electronic medical records: a prevalence and validation study using the Deliver Primary Healthcare Information (DELPHI) database
Source: BMC Musculoskelet Disord. 2019 May 3;20:187. doi: 10.1186/s12891-019-2568-2 (PMC6499985; doi:10.1186/s12891-019-2568-2)
Supplement: Supplementary file 1 — Table S1. Prevalence of the Top 20 ICPC Coded Musculoskeletal Conditions (n = 2493 patients). Table S2. Prevalence of the Top 20 ICD9 Coded Musculoskeletal Conditions (n = 2493 patients) (DOCX 14 kb) [file 12891_2019_2568_MOESM1_ESM.docx]

**Table S1. Prevalence of the Top 20 ICPC Coded Musculoskeletal Conditions (n=2493 patients)**

|  |  | **# Patients** | **% Patients** |
| --- | --- | --- | --- |
| 1 | L90 Osteoarthrosis of knee | 164 | 6.6% |
| 2 | L08 Shoulder symptom/complaint | 155 | 6.2% |
| 3 | L02 Back symptom/complaint | 149 | 6.0% |
| 4 | L84 Back syndrome without radiating pain | 146 | 5.9% |
| 5 | L15 Knee symptom/complaint | 144 | 5.8% |
| 6 | L17 Foot/toe symptom/complaint | 144 | 5.8% |
| 7 | L91 Osteoarthrosis other | 142 | 5.7% |
| 8 | L86 Back syndrome with radiating pain | 115 | 4.6% |
| 9 | L95 Osteoporosis | 112 | 4.5% |
| 10 | L92 Shoulder syndrome | 108 | 4.3% |
| 11 | L01 Neck symptom/complaint | 103 | 4.1% |
| 12 | L89 Osteoarthrosis of hip | 93 | 3.7% |
| 13 | L13 Hip symptom/complaint | 82 | 3.3% |
| 14 | L14 Leg/thigh symptom/complaint | 77 | 3.1% |
| 15 | L12 Hand/finger symptom/complaint | 74 | 3.0% |
| 16 | L99 Musculoskeletal disease other | 74 | 3.0% |
| 17 | L83 Neck syndrome | 70 | 2.8% |
| 18 | L87 Bursitis/tendinitis/synovitis NOS | 65 | 2.6% |
| 19 | L18 Muscle pain | 61 | 2.4% |
| 20 | L03 Low back symptom/complaint | 57 | 2.3% |

Note: Percentages will not add to 100% because patients can have 2+ conditions.

**Table S2. Prevalence of the Top 20 ICD9 Coded Musculoskeletal Conditions (n=2493 patients)**

|  |  | **# Patients** | **% Patients** |
| --- | --- | --- | --- |
| 1 | 715 Osteoarthritis | 194 | 7.8% |
| 2 | 724 Lumbar strain, lumbago, coccydynia, sciatica | 180 | 7.2% |
| 3 | 727 Synovitis, tenosynovitis, bursitis, bunion, ganglion | 170 | 6.8% |
| 4 | 781 Leg cramps, leg pain, muscle pain, joint pain, arthralgia, joint swelling, masses | 159 | 6.4% |
| 5 | 844 Knee pain/Sprain (leg/knee) | 44 | 1.8% |
| 6 | 847 Cervical disc disease/Sprain (neck) | 43 | 1.7% |
| 7 | 840 Sprain (shoulder) | 37 | 1.5% |
| 8 | 733 Osteoporosis, spontaneous fracture, other disorders of bone and cartilage | 34 | 1.4% |
| 9 | 739 Other diseases of musculoskeletal system and connective tissue | 25 | 1.0% |
| 10 | 845 Ankle strain/Sprain (foot/ankle) | 25 | 1.0% |
| 11 | 722 Intervertebral disc disorders | 22 | 0.9% |
| 12 | 274 Gout | 19 | 0.8% |
| 13 | 729 Fibrositis, myositis, muscular rheumatism | 15 | 0.6% |
| 14 | 842 Sprain (wrist) | 12 | 0.5% |
| 15 | 714 Rheumatoid arthritis, Still's disease | 10 | 0.4% |
| 16 | 370 Joint - Keratitis | 9 | 0.4% |
| 17 | 700 Calluses | 9 | 0.4% |
| 18 | 703 Ingrown Nail | 8 | 0.3% |
| 19 | 701 Joint - Keloid | 6 | 0.2% |
| 20 | 710 Disseminated lupus erythematosus, generalized scleroderma, dermatomyositis, polymyositis | 6 | 0.2% |

Note: Percentages will not add to 100% because patients can have 2+ conditions.
