# Supplementary material for: Identifying musculoskeletal conditions in electronic medical records: a prevalence and validation study using the Deliver Primary Healthcare Information (DELPHI) database
Source: BMC Musculoskelet Disord. 2019 May 3;20:187. doi: 10.1186/s12891-019-2568-2 (PMC6499985; doi:10.1186/s12891-019-2568-2)
Supplement: Supplementary file 2 — Table S3. Most Frequent Referrals at ICPC Reference Standard Musculoskeletal Coded Encounters Compared to Non Musculoskeletal Encounters (DOCX 13 kb) [file 12891_2019_2568_MOESM2_ESM.docx]

**Table S3. Most Frequent Referrals at ICPC Reference Standard Musculoskeletal Coded Encounters Compared to Non Musculoskeletal Encounters**

|  | **ICPC Reference Standard Coded Musculoskeletal Encounters (n=5160)** | | **Non Musculoskeletal Encounters (n=16804)** | |
| --- | --- | --- | --- | --- |
|  | **#** | **%** | **#** | **#** |
| Orthopedic surgery | 260 | 5.0% | 93 | 0.6% |
| Dermatology | 113 | 2.2% | 426 | 2.5% |
| General Surgery | 76 | 1.5% | 240 | 1.4% |
| Internal Medicine | 67 | 1.3% | 146 | 0.9% |
| Specialty clinic | 53 | 1.0% | 68 | 0.4% |
| Neurology | 43 | 0.8% | 101 | 0.6% |
| Urology | 40 | 0.8% | 141 | 0.8% |
| Plastic Surgery | 34 | 0.7% | 67 | 0.4% |
| Rheumatology | 33 | 0.6% | 22 | 0.1% |
| Ears/Nose/Throat | 31 | 0.6% | 130 | 0.8% |
| General Practice | 31 | 0.6% | 94 | 0.6% |
| OBGYN | 31 | 0.6% | 70 | 0.4% |
| Gastroenterology | 30 | 0.6% | 160 | 1.0% |
| Cardiology | 20 | 0.4% | 124 | 0.7% |
| Geriatrics | 14 | 0.3% | 35 | 0.2% |
| Endocrinology | 13 | 0.3% | 52 | 0.3% |
| Respirology | 12 | 0.2% | 47 | 0.3% |
| Hematology | 8 | 0.2% | 16 | 0.1% |
| Optometry | 8 | 0.2% | 5 | 0.0% |
| Psychiatry | 7 | 0.1% | 90 | 0.5% |
| Allergists | 5 | 0.1% | 23 | 0.1% |
| Vascular Surgery | 5 | 0.1% | 30 | 0.2% |
| Other | 44 | 0.9% | 48 | 0.3% |

Note: will not add to 100% because some encounters had more than one referral.

Referrals to physiotherapy do not require a referral and are not included.
